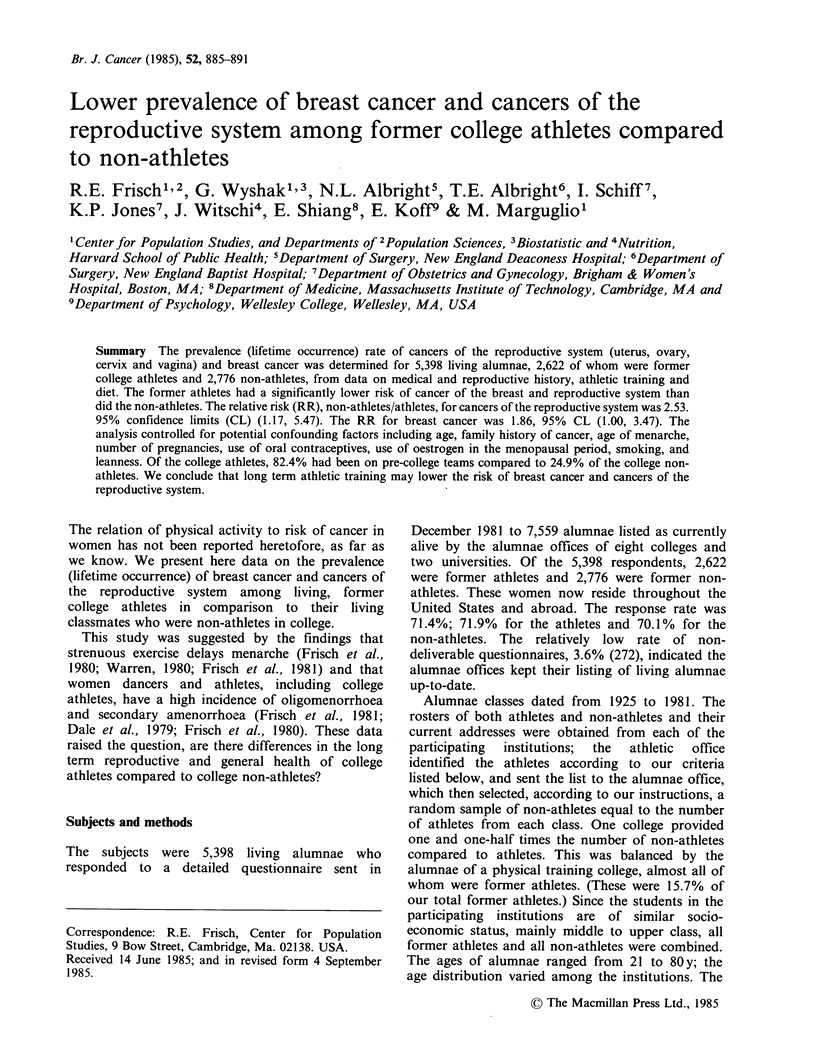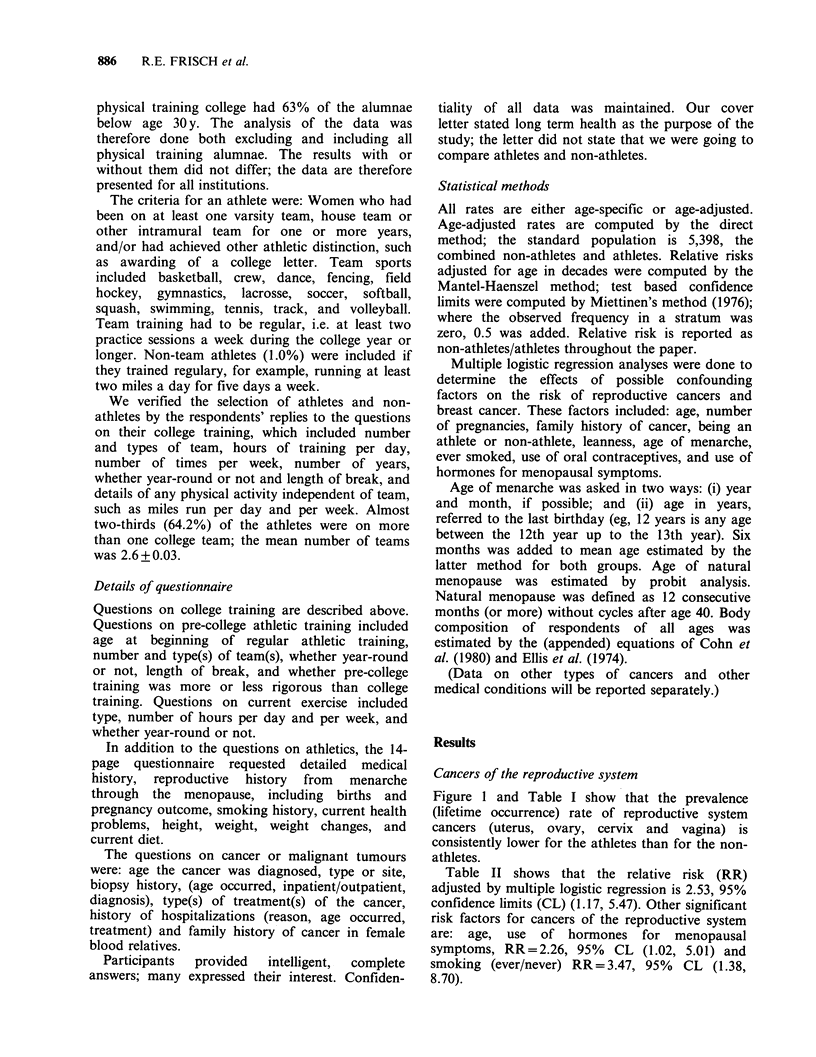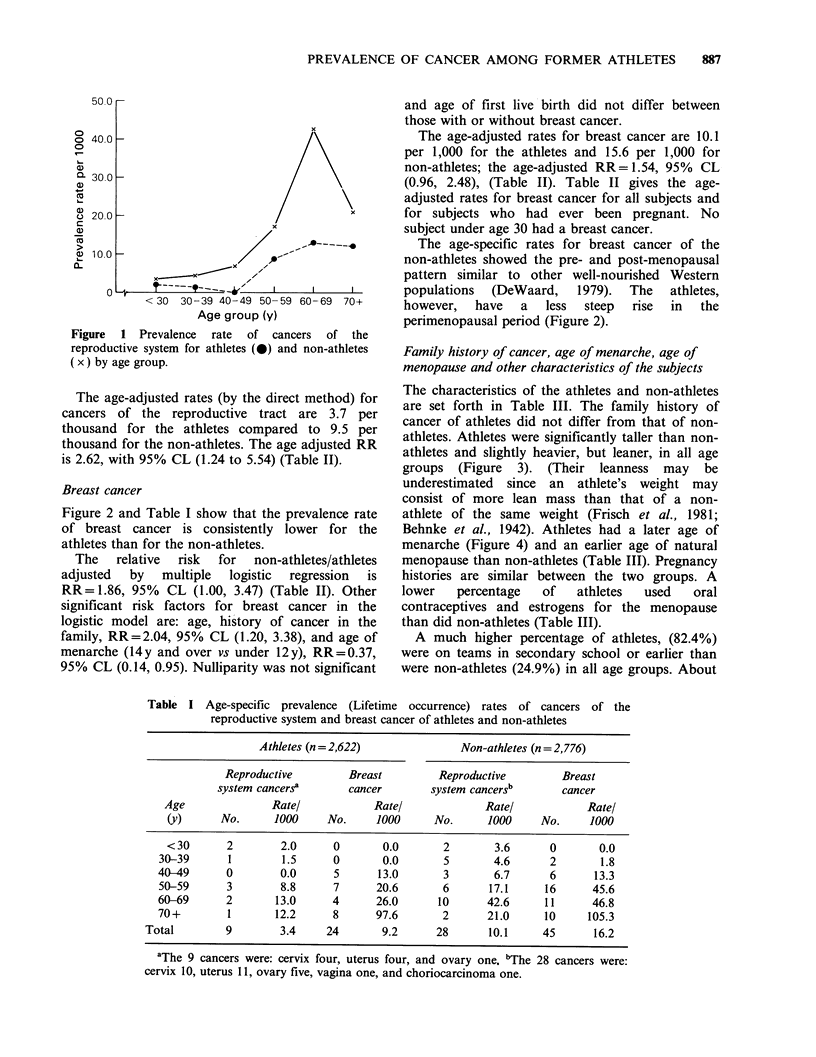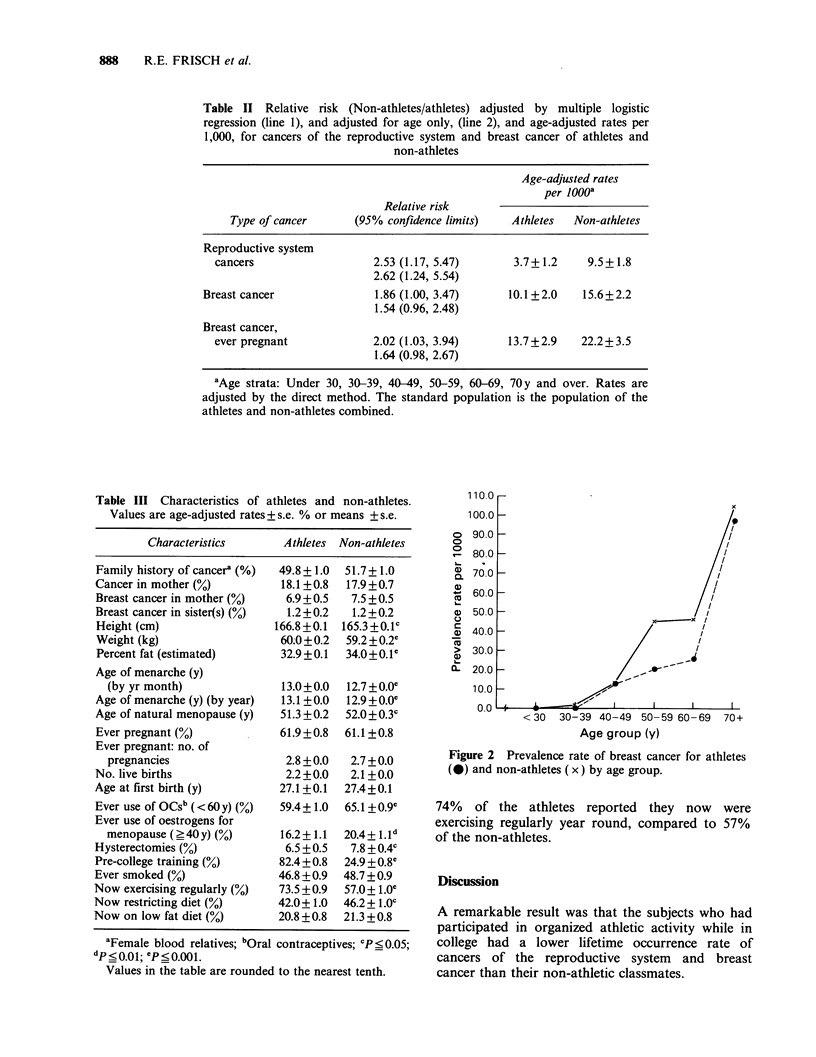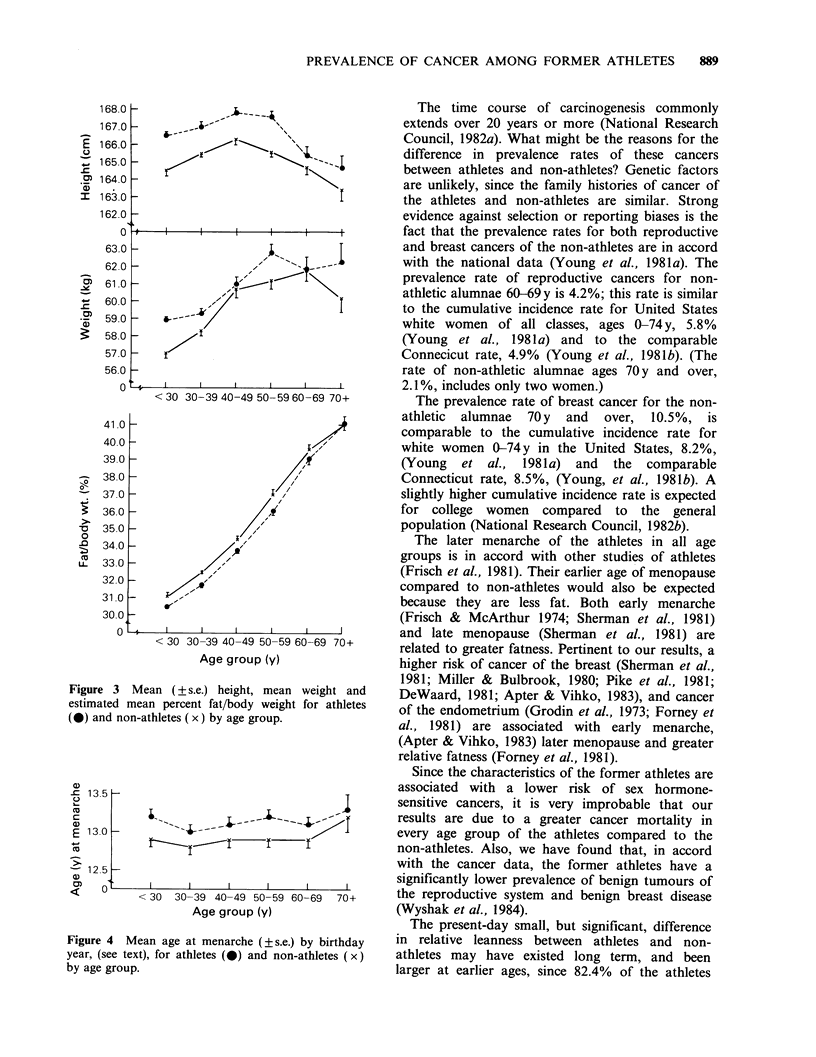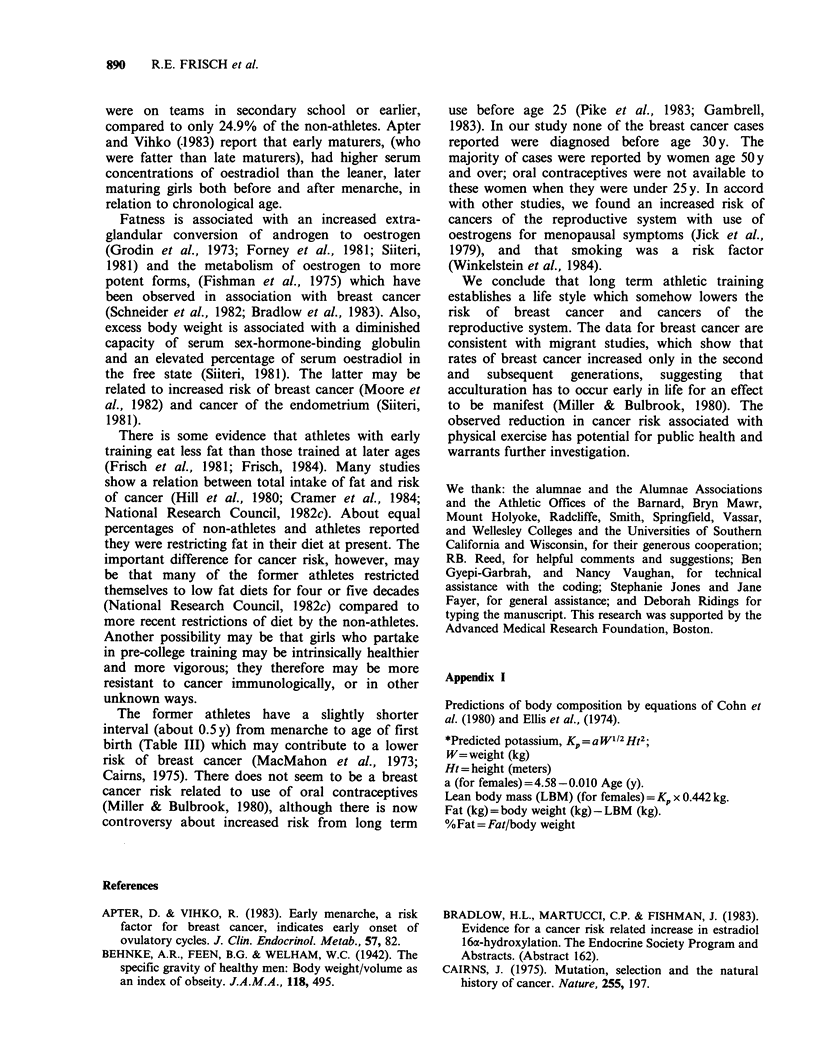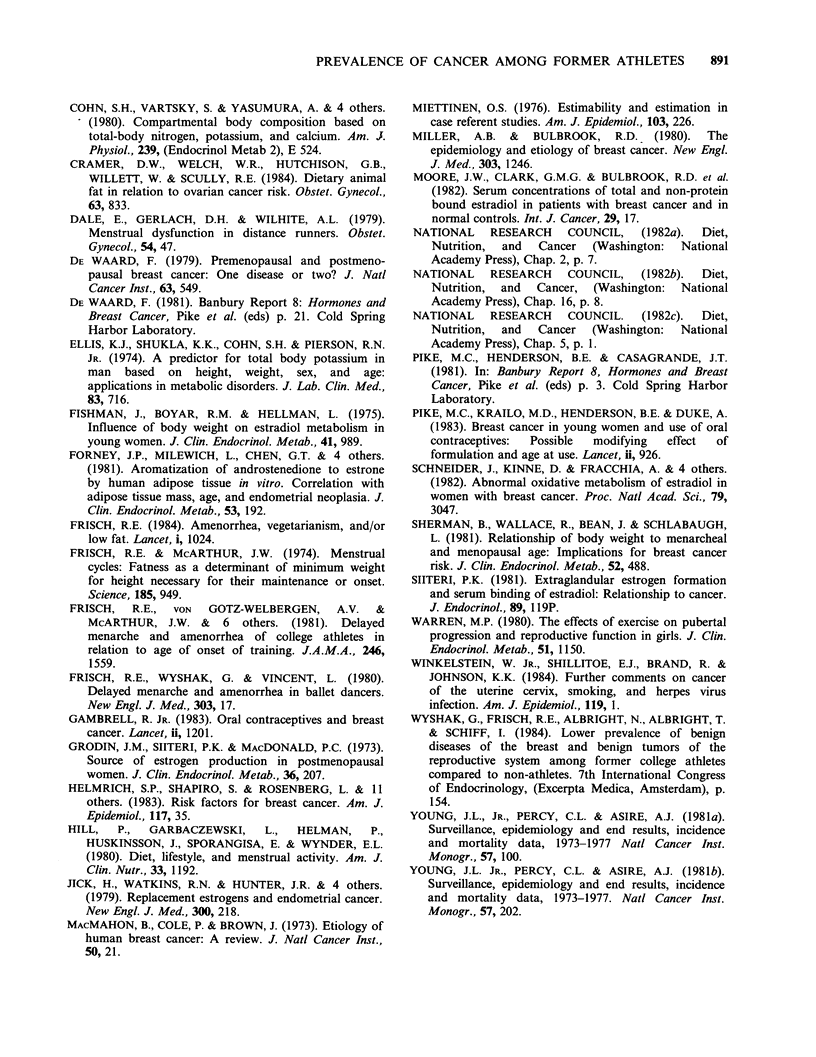# Lower prevalence of breast cancer and cancers of the reproductive system among former college athletes compared to non-athletes.

**DOI:** 10.1038/bjc.1985.273

**Published:** 1985-12

**Authors:** R. E. Frisch, G. Wyshak, N. L. Albright, T. E. Albright, I. Schiff, K. P. Jones, J. Witschi, E. Shiang, E. Koff, M. Marguglio

## Abstract

The prevalence (lifetime occurrence) rate of cancers of the reproductive system (uterus, ovary, cervix and vagina) and breast cancer was determined for 5,398 living alumnae, 2,622 of whom were former college athletes and 2,776 non-athletes, from data on medical and reproductive history, athletic training and diet. The former athletes had a significantly lower risk of cancer of the breast and reproductive system than did the non-athletes. The relative risk (RR), non-athletes/athletes, for cancers of the reproductive system was 2.53. 95% confidence limits (CL) (1.17, 5.47). The RR for breast cancer was 1.86, 95% CL (1.00, 3.47). The analysis controlled for potential confounding factors including age, family history of cancer, age of menarche, number of pregnancies, use of oral contraceptives, use of oestrogen in the menopausal period, smoking, and leanness. Of the college athletes, 82.4% had been on pre-college teams compared to 24.9% of the college non-athletes. We conclude that long term athletic training may lower the risk of breast cancer and cancers of the reproductive system.


*Br. J. Cancer* (1985), **52**, 885–891

### Lower prevalence of breast cancer and cancers of the reproductive system among former college athletes compared to non-athletes

R.E. Frisch[1,2], G. Wyshak[1,3], N.L. Albright[5], T.E. Albright[6], I. Schiff[7], K.P. Jones[7], J. Witschi[4], E. Shiang[8], E. Koff[9] & M. Marguglio[1]

[1]*Center for Population Studies, and Departments of* [2]*Population Sciences,* [3]*Biostatistic and* [4]*Nutrition, Harvard School of Public Health;* [5]*Department of Surgery, New England Deaconess Hospital;* [6]*Department of Surgery, New England Baptist Hospital;* [7]*Department of Obstetrics and Gynecology, Brigham & Women's Hospital, Boston, MA;* [8]*Department of Medicine, Massachusetts Institute of Technology, Cambridge, MA and* [9]*Department of Psychology, Wellesley College, Wellesley, MA, USA*

**Summary** The prevalence (lifetime occurrence) rate of cancers of the reproductive system (uterus, ovary, cervix and vagina) and breast cancer was determined for 5,398 living alumnae, 2,622 of whom were former college athletes and 2,776 non-athletes, from data on medical and reproductive history, athletic training and diet. The former athletes had a significantly lower risk of cancer of the breast and reproductive system than did the non-athletes. The relative risk (RR), non-athletes/athletes, for cancers of the reproductive system was 2.53. 95% confidence limits (CL) (1.17, 5.47). The RR for breast cancer was 1.86, 95% CL (1.00, 3.47). The analysis controlled for potential confounding factors including age, family history of cancer, age of menarche, number of pregnancies, use of oral contraceptives, use of oestrogen in the menopausal period, smoking, and leanness. Of the college athletes, 82.4% had been on pre-college teams compared to 24.9% of the college non-athletes. We conclude that long term athletic training may lower the risk of breast cancer and cancers of the reproductive system.

The relation of physical activity to risk of cancer in women has not been reported heretofore, as far as we know. We present here data on the prevalence (lifetime occurrence) of breast cancer and cancers of the reproductive system among living, former college athletes in comparison to their living classmates who were non-athletes in college.

This study was suggested by the findings that strenuous exercise delays menarche (Frisch *et al.*, 1980; Warren, 1980; Frisch *et al.*, 1981) and that women dancers and athletes, including college athletes, have a high incidence of oligomenorrhoea and secondary amenorrhoea (Frisch *et al.*, 1981; Dale *et al.*, 1979; Frisch *et al.*, 1980). These data raised the question, are there differences in the long term reproductive and general health of college athletes compared to college non-athletes?

### Subjects and methods

The subjects were 5,398 living alumnae who responded to a detailed questionnaire sent in December 1981 to 7,559 alumnae listed as currently alive by the alumnae offices of eight colleges and two universities. Of the 5,398 respondents, 2,622 were former athletes and 2,776 were former non-athletes. These women now reside throughout the United States and abroad. The response rate was 71.4%; 71.9% for the athletes and 70.1% for the non-athletes. The relatively low rate of non-deliverable questionnaires, 3.6% (272), indicated the alumnae offices kept their listing of living alumnae up-to-date.

Alumnae classes dated from 1925 to 1981. The rosters of both athletes and non-athletes and their current addresses were obtained from each of the participating institutions; the athletic office identified the athletes according to our criteria listed below, and sent the list to the alumnae office, which then selected, according to our instructions, a random sample of non-athletes equal to the number of athletes from each class. One college provided one and one-half times the number of non-athletes compared to athletes. This was balanced by the alumnae of a physical training college, almost all of whom were former athletes. (These were 15.7% of our total former athletes.) Since the students in the participating institutions are of similar socio-economic status, mainly middle to upper class, all former athletes and all non-athletes were combined. The ages of alumnae ranged from 21 to 80 y; the age distribution varied among the institutions. The

Correspondence: R.E. Frisch, Center for Population Studies, 9 Bow Street, Cambridge, Ma. 02138, USA.
Received 14 June 1985; and in revised form 4 September 1985.

© The Macmillan Press Ltd., 1985

physical training college had 63% of the alumnae below age 30 y. The analysis of the data was therefore done both excluding and including all physical training alumnae. The results with or without them did not differ; the data are therefore presented for all institutions.

The criteria for an athlete were: Women who had been on at least one varsity team, house team or other intramural team for one or more years, and/or had achieved other athletic distinction, such as awarding of a college letter. Team sports included basketball, crew, dance, fencing, field hockey, gymnastics, lacrosse, soccer, softball, squash, swimming, tennis, track, and volleyball. Team training had to be regular, i.e. at least two practice sessions a week during the college year or longer. Non-team athletes (1.0%) were included if they trained regulary, for example, running at least two miles a day for five days a week.

We verified the selection of athletes and non-athletes by the respondents' replies to the questions on their college training, which included number and types of team, hours of training per day, number of times per week, number of years, whether year-round or not and length of break, and details of any physical activity independent of team, such as miles run per day and per week. Almost two-thirds (64.2%) of the athletes were on more than one college team; the mean number of teams was $2.6 \pm 0.03$.

*Details of questionnaire*

Questions on college training are described above. Questions on pre-college athletic training included age at beginning of regular athletic training, number and type(s) of team(s), whether year-round or not, length of break, and whether pre-college training was more or less rigorous than college training. Questions on current exercise included type, number of hours per day and per week, and whether year-round or not.

In addition to the questions on athletics, the 14-page questionnaire requested detailed medical history, reproductive history from menarche through the menopause, including births and pregnancy outcome, smoking history, current health problems, height, weight, weight changes, and current diet.

The questions on cancer or malignant tumours were: age the cancer was diagnosed, type or site, biopsy history, (age occurred, inpatient/outpatient, diagnosis), type(s) of treatment(s) of the cancer, history of hospitalizations (reason, age occurred, treatment) and family history of cancer in female blood relatives.

Participants provided intelligent, complete answers; many expressed their interest. Confidentiality of all data was maintained. Our cover letter stated long term health as the purpose of the study; the letter did not state that we were going to compare athletes and non-athletes.

*Statistical methods*

All rates are either age-specific or age-adjusted. Age-adjusted rates are computed by the direct method; the standard population is 5,398, the combined non-athletes and athletes. Relative risks adjusted for age in decades were computed by the Mantel-Haenszel method; test based confidence limits were computed by Miettinen's method (1976); where the observed frequency in a stratum was zero, 0.5 was added. Relative risk is reported as non-athletes/athletes throughout the paper.

Multiple logistic regression analyses were done to determine the effects of possible confounding factors on the risk of reproductive cancers and breast cancer. These factors included: age, number of pregnancies, family history of cancer, being an athlete or non-athlete, leanness, age of menarche, ever smoked, use of oral contraceptives, and use of hormones for menopausal symptoms.

Age of menarche was asked in two ways: (i) year and month, if possible; and (ii) age in years, referred to the last birthday (eg, 12 years is any age between the 12th year up to the 13th year). Six months was added to mean age estimated by the latter method for both groups. Age of natural menopause was estimated by probit analysis. Natural menopause was defined as 12 consecutive months (or more) without cycles after age 40. Body composition of respondents of all ages was estimated by the (appended) equations of Cohn *et al.* (1980) and Ellis *et al.* (1974).

(Data on other types of cancers and other medical conditions will be reported separately.)

### Results

*Cancers of the reproductive system*

Figure 1 and Table I show that the prevalence (lifetime occurrence) rate of reproductive system cancers (uterus, ovary, cervix and vagina) is consistently lower for the athletes than for the non-athletes.

Table II shows that the relative risk (RR) adjusted by multiple logistic regression is 2.53, 95% confidence limits (CL) (1.17, 5.47). Other significant risk factors for cancers of the reproductive system are: age, use of hormones for menopausal symptoms, RR = 2.26, 95% CL (1.02, 5.01) and smoking (ever/never) RR = 3.47, 95% CL (1.38, 8.70).

**Figure 1** Prevalence rate of cancers of the reproductive system for athletes (●) and non-athletes (×) by age group.

The age-adjusted rates (by the direct method) for cancers of the reproductive tract are 3.7 per thousand for the athletes compared to 9.5 per thousand for the non-athletes. The age adjusted RR is 2.62, with 95% CL (1.24 to 5.54) (Table II).

*Breast cancer*

Figure 2 and Table I show that the prevalence rate of breast cancer is consistently lower for the athletes than for the non-athletes.

The relative risk for non-athletes/athletes adjusted by multiple logistic regression is RR = 1.86, 95% CL (1.00, 3.47) (Table II). Other significant risk factors for breast cancer in the logistic model are: age, history of cancer in the family, RR = 2.04, 95% CL (1.20, 3.38), and age of menarche (14 y and over *vs* under 12 y), RR = 0.37, 95% CL (0.14, 0.95). Nulliparity was not significant and age of first live birth did not differ between those with or without breast cancer.

The age-adjusted rates for breast cancer are 10.1 per 1,000 for the athletes and 15.6 per 1,000 for non-athletes; the age-adjusted RR = 1.54, 95% CL (0.96, 2.48), (Table II). Table II gives the age-adjusted rates for breast cancer for all subjects and for subjects who had ever been pregnant. No subject under age 30 had a breast cancer.

The age-specific rates for breast cancer of the non-athletes showed the pre- and post-menopausal pattern similar to other well-nourished Western populations (DeWaard, 1979). The athletes, however, have a less steep rise in the perimenopausal period (Figure 2).

*Family history of cancer, age of menarche, age of menopause and other characteristics of the subjects*

The characteristics of the athletes and non-athletes are set forth in Table III. The family history of cancer of athletes did not differ from that of non-athletes. Athletes were significantly taller than non-athletes and slightly heavier, but leaner, in all age groups (Figure 3). (Their leanness may be underestimated since an athlete's weight may consist of more lean mass than that of a non-athlete of the same weight (Frisch *et al.*, 1981; Behnke *et al.*, 1942). Athletes had a later age of menarche (Figure 4) and an earlier age of natural menopause than non-athletes (Table III). Pregnancy histories are similar between the two groups. A lower percentage of athletes used oral contraceptives and estrogens for the menopause than did non-athletes (Table III).

A much higher percentage of athletes, (82.4%) were on teams in secondary school or earlier than were non-athletes (24.9%) in all age groups. About

**Table I** Age-specific prevalence (Lifetime occurrence) rates of cancers of the reproductive system and breast cancer of athletes and non-athletes

| | *Athletes (n = 2,622)* | | | | *Non-athletes (n = 2,776)* | | | |
|---|---|---|---|---|---|---|---|---|
| | *Reproductive system cancers*[a] | | *Breast cancer* | | *Reproductive system cancers*[b] | | *Breast cancer* | |
| *Age (y)* | *No.* | *Rate/1000* | *No.* | *Rate/1000* | *No.* | *Rate/1000* | *No.* | *Rate/1000* |
| <30 | 2 | 2.0 | 0 | 0.0 | 2 | 3.6 | 0 | 0.0 |
| 30–39 | 1 | 1.5 | 0 | 0.0 | 5 | 4.6 | 2 | 1.8 |
| 40–49 | 0 | 0.0 | 5 | 13.0 | 3 | 6.7 | 6 | 13.3 |
| 50–59 | 3 | 8.8 | 7 | 20.6 | 6 | 17.1 | 16 | 45.6 |
| 60–69 | 2 | 13.0 | 4 | 26.0 | 10 | 42.6 | 11 | 46.8 |
| 70+ | 1 | 12.2 | 8 | 97.6 | 2 | 21.0 | 10 | 105.3 |
| Total | 9 | 3.4 | 24 | 9.2 | 28 | 10.1 | 45 | 16.2 |

[a]The 9 cancers were: cervix four, uterus four, and ovary one. [b]The 28 cancers were: cervix 10, uterus 11, ovary five, vagina one, and choriocarcinoma one.

**Table II** Relative risk (Non-athletes/athletes) adjusted by multiple logistic regression (line 1), and adjusted for age only, (line 2), and age-adjusted rates per 1,000, for cancers of the reproductive system and breast cancer of athletes and non-athletes

| *Type of cancer* | *Relative risk (95% confidence limits)* | *Age-adjusted rates per 1000*[a] *Athletes* | *Non-athletes* |
|---|---|---|---|
| Reproductive system cancers | 2.53 (1.17, 5.47)<br>2.62 (1.24, 5.54) | 3.7±1.2 | 9.5±1.8 |
| Breast cancer | 1.86 (1.00, 3.47)<br>1.54 (0.96, 2.48) | 10.1±2.0 | 15.6±2.2 |
| Breast cancer, ever pregnant | 2.02 (1.03, 3.94)<br>1.64 (0.98, 2.67) | 13.7±2.9 | 22.2±3.5 |

[a]Age strata: Under 30, 30–39, 40–49, 50–59, 60–69, 70 y and over. Rates are adjusted by the direct method. The standard population is the population of the athletes and non-athletes combined.

**Table III** Characteristics of athletes and non-athletes. Values are age-adjusted rates±s.e. % or means ±s.e.

| *Characteristics* | *Athletes* | *Non-athletes* |
|---|---|---|
| Family history of cancer[a] (%) | 49.8±1.0 | 51.7±1.0 |
| Cancer in mother (%) | 18.1±0.8 | 17.9±0.7 |
| Breast cancer in mother (%) | 6.9±0.5 | 7.5±0.5 |
| Breast cancer in sister(s) (%) | 1.2±0.2 | 1.2±0.2 |
| Height (cm) | 166.8±0.1 | 165.3±0.1[c] |
| Weight (kg) | 60.0±0.2 | 59.2±0.2[e] |
| Percent fat (estimated) | 32.9±0.1 | 34.0±0.1[e] |
| Age of menarche (y) (by yr month) | 13.0±0.0 | 12.7±0.0[e] |
| Age of menarche (y) (by year) | 13.1±0.0 | 12.9±0.0[e] |
| Age of natural menopause (y) | 51.3±0.2 | 52.0±0.3[c] |
| Ever pregnant (%) | 61.9±0.8 | 61.1±0.8 |
| Ever pregnant: no. of pregnancies | 2.8±0.0 | 2.7±0.0 |
| No. live births | 2.2±0.0 | 2.1±0.0 |
| Age at first birth (y) | 27.1±0.1 | 27.4±0.1 |
| Ever use of OCs[b] (<60 y) (%) | 59.4±1.0 | 65.1±0.9[e] |
| Ever use of oestrogens for menopause (≥40 y) (%) | 16.2±1.1 | 20.4±1.1[d] |
| Hysterectomies (%) | 6.5±0.5 | 7.8±0.4[c] |
| Pre-college training (%) | 82.4±0.8 | 24.9±0.8[e] |
| Ever smoked (%) | 46.8±0.9 | 48.7±0.9 |
| Now exercising regularly (%) | 73.5±0.9 | 57.0±1.0[e] |
| Now restricting diet (%) | 42.0±1.0 | 46.2±1.0[c] |
| Now on low fat diet (%) | 20.8±0.8 | 21.3±0.8 |

[a]Female blood relatives; [b]Oral contraceptives; [c]$P \leq 0.05$; [d]$P \leq 0.01$; [e]$P \leq 0.001$.

Values in the table are rounded to the nearest tenth.

**Figure 2** Prevalence rate of breast cancer for athletes (●) and non-athletes (×) by age group.

74% of the athletes reported they now were exercising regularly year round, compared to 57% of the non-athletes.

### Discussion

A remarkable result was that the subjects who had participated in organized athletic activity while in college had a lower lifetime occurrence rate of cancers of the reproductive system and breast cancer than their non-athletic classmates.

**Figure 3** Mean (±s.e.) height, mean weight and estimated mean percent fat/body weight for athletes (●) and non-athletes (×) by age group.

**Figure 4** Mean age at menarche (±s.e.) by birthday year, (see text), for athletes (●) and non-athletes (×) by age group.

The time course of carcinogenesis commonly extends over 20 years or more (National Research Council, 1982*a*). What might be the reasons for the difference in prevalence rates of these cancers between athletes and non-athletes? Genetic factors are unlikely, since the family histories of cancer of the athletes and non-athletes are similar. Strong evidence against selection or reporting biases is the fact that the prevalence rates for both reproductive and breast cancers of the non-athletes are in accord with the national data (Young *et al.*, 1981*a*). The prevalence rate of reproductive cancers for non-athletic alumnae 60–69 y is 4.2%; this rate is similar to the cumulative incidence rate for United States white women of all classes, ages 0–74 y, 5.8% (Young *et al.*, 1981*a*) and to the comparable Connecicut rate, 4.9% (Young *et al.*, 1981*b*). (The rate of non-athletic alumnae ages 70 y and over, 2.1%, includes only two women.)

The prevalence rate of breast cancer for the non-athletic alumnae 70 y and over, 10.5%, is comparable to the cumulative incidence rate for white women 0–74 y in the United States, 8.2%, (Young *et al.*, 1981*a*) and the comparable Connecticut rate, 8.5%, (Young, *et al.*, 1981*b*). A slightly higher cumulative incidence rate is expected for college women compared to the general population (National Research Council, 1982*b*).

The later menarche of the athletes in all age groups is in accord with other studies of athletes (Frisch *et al.*, 1981). Their earlier age of menopause compared to non-athletes would also be expected because they are less fat. Both early menarche (Frisch & McArthur 1974; Sherman *et al.*, 1981) and late menopause (Sherman *et al.*, 1981) are related to greater fatness. Pertinent to our results, a higher risk of cancer of the breast (Sherman *et al.*, 1981; Miller & Bulbrook, 1980; Pike *et al.*, 1981; DeWaard, 1981; Apter & Vihko, 1983), and cancer of the endometrium (Grodin *et al.*, 1973; Forney *et al.*, 1981) are associated with early menarche, (Apter & Vihko, 1983) later menopause and greater relative fatness (Forney *et al.*, 1981).

Since the characteristics of the former athletes are associated with a lower risk of sex hormone-sensitive cancers, it is very improbable that our results are due to a greater cancer mortality in every age group of the athletes compared to the non-athletes. Also, we have found that, in accord with the cancer data, the former athletes have a significantly lower prevalence of benign tumours of the reproductive system and benign breast disease (Wyshak *et al.*, 1984).

The present-day small, but significant, difference in relative leanness between athletes and non-athletes may have existed long term, and been larger at earlier ages, since 82.4% of the athletes were on teams in secondary school or earlier, compared to only 24.9% of the non-athletes. Apter and Vihko (1983) report that early maturers, (who were fatter than late maturers), had higher serum concentrations of oestradiol than the leaner, later maturing girls both before and after menarche, in relation to chronological age.

Fatness is associated with an increased extra-glandular conversion of androgen to oestrogen (Grodin *et al.*, 1973; Forney *et al.*, 1981; Siiteri, 1981) and the metabolism of oestrogen to more potent forms, (Fishman *et al.*, 1975) which have been observed in association with breast cancer (Schneider *et al.*, 1982; Bradlow *et al.*, 1983). Also, excess body weight is associated with a diminished capacity of serum sex-hormone-binding globulin and an elevated percentage of serum oestradiol in the free state (Siiteri, 1981). The latter may be related to increased risk of breast cancer (Moore *et al.*, 1982) and cancer of the endometrium (Siiteri, 1981).

There is some evidence that athletes with early training eat less fat than those trained at later ages (Frisch *et al.*, 1981; Frisch, 1984). Many studies show a relation between total intake of fat and risk of cancer (Hill *et al.*, 1980; Cramer *et al.*, 1984; National Research Council, 1982*c*). About equal percentages of non-athletes and athletes reported they were restricting fat in their diet at present. The important difference for cancer risk, however, may be that many of the former athletes restricted themselves to low fat diets for four or five decades (National Research Council, 1982*c*) compared to more recent restrictions of diet by the non-athletes. Another possibility may be that girls who partake in pre-college training may be intrinsically healthier and more vigorous; they therefore may be more resistant to cancer immunologically, or in other unknown ways.

The former athletes have a slightly shorter interval (about 0.5 y) from menarche to age of first birth (Table III) which may contribute to a lower risk of breast cancer (MacMahon *et al.*, 1973; Cairns, 1975). There does not seem to be a breast cancer risk related to use of oral contraceptives (Miller & Bulbrook, 1980), although there is now controversy about increased risk from long term use before age 25 (Pike *et al.*, 1983; Gambrell, 1983). In our study none of the breast cancer cases reported were diagnosed before age 30 y. The majority of cases were reported by women age 50 y and over; oral contraceptives were not available to these women when they were under 25 y. In accord with other studies, we found an increased risk of cancers of the reproductive system with use of oestrogens for menopausal symptoms (Jick *et al.*, 1979), and that smoking was a risk factor (Winkelstein *et al.*, 1984).

We conclude that long term athletic training establishes a life style which somehow lowers the risk of breast cancer and cancers of the reproductive system. The data for breast cancer are consistent with migrant studies, which show that rates of breast cancer increased only in the second and subsequent generations, suggesting that acculturation has to occur early in life for an effect to be manifest (Miller & Bulbrook, 1980). The observed reduction in cancer risk associated with physical exercise has potential for public health and warrants further investigation.

We thank: the alumnae and the Alumnae Associations and the Athletic Offices of the Barnard, Bryn Mawr, Mount Holyoke, Radcliffe, Smith, Springfield, Vassar, and Wellesley Colleges and the Universities of Southern California and Wisconsin, for their generous cooperation; RB. Reed, for helpful comments and suggestions; Ben Gyepi-Garbrah, and Nancy Vaughan, for technical assistance with the coding; Stephanie Jones and Jane Fayer, for general assistance; and Deborah Ridings for typing the manuscript. This research was supported by the Advanced Medical Research Foundation, Boston.

### Appendix I

Predictions of body composition by equations of Cohn *et al.* (1980) and Ellis *et al.*, (1974).

*Predicted potassium, $K_p = aW^{1/2}Ht^2$;
$W$ = weight (kg)
$Ht$ = height (meters)
a (for females) = 4.58 − 0.010 Age (y).
Lean body mass (LBM) (for females) = $K_p \times 0.442$ kg.
Fat (kg) = body weight (kg) − LBM (kg).
%Fat = *Fat*/body weight

### References

APTER, D. & VIHKO, R. (1983). Early menarche, a risk factor for breast cancer, indicates early onset of ovulatory cycles. *J. Clin. Endocrinol. Metab.*, **57**, 82.

BEHNKE, A.R., FEEN, B.G. & WELHAM, W.C. (1942). The specific gravity of healthy men: Body weight/volume as an index of obseity. *J.A.M.A.*, **118**, 495.

BRADLOW, H.L., MARTUCCI, C.P. & FISHMAN, J. (1983). Evidence for a cancer risk related increase in estradiol 16α-hydroxylation. The Endocrine Society Program and Abstracts. (Abstract 162).

CAIRNS, J. (1975). Mutation, selection and the natural history of cancer. *Nature*, **255**, 197.

COHN, S.H., VARTSKY, S. & YASUMURA, A. & 4 others. (1980). Compartmental body composition based on total-body nitrogen, potassium, and calcium. *Am. J. Physiol.*, **239**, (Endocrinol Metab 2), E 524.

CRAMER, D.W., WELCH, W.R., HUTCHISON, G.B., WILLETT, W. & SCULLY, R.E. (1984). Dietary animal fat in relation to ovarian cancer risk. *Obstet. Gynecol.*, **63**, 833.

DALE, E., GERLACH, D.H. & WILHITE, A.L. (1979). Menstrual dysfunction in distance runners. *Obstet. Gynecol.*, **54**, 47.

DE WAARD, F. (1979). Premenopausal and postmenopausal breast cancer: One disease or two? *J. Natl Cancer Inst.*, **63**, 549.

DE WAARD, F. (1981). Banbury Report 8: *Hormones and Breast Cancer*, Pike *et al.* (eds) p. 21. Cold Spring Harbor Laboratory.

ELLIS, K.J., SHUKLA, K.K., COHN, S.H. & PIERSON, R.N. JR. (1974). A predictor for total body potassium in man based on height, weight, sex, and age: applications in metabolic disorders. *J. Lab. Clin. Med.*, **83**, 716.

FISHMAN, J., BOYAR, R.M. & HELLMAN, L. (1975). Influence of body weight on estradiol metabolism in young women. *J. Clin. Endocrinol. Metab.*, **41**, 989.

FORNEY, J.P., MILEWICH, L., CHEN, G.T. & 4 others. (1981). Aromatization of androstenedione to estrone by human adipose tissue *in vitro*. Correlation with adipose tissue mass, age, and endometrial neoplasia. *J. Clin. Endocrinol. Metab.*, **53**, 192.

FRISCH, R.E. (1984). Amenorrhea, vegetarianism, and/or low fat. *Lancet*, **i**, 1024.

FRISCH, R.E. & MCARTHUR, J.W. (1974). Menstrual cycles: Fatness as a determinant of minimum weight for height necessary for their maintenance or onset. *Science*, **185**, 949.

FRISCH, R.E., VON GOTZ-WELBERGEN, A.V. & MCARTHUR, J.W. & 6 others. (1981). Delayed menarche and amenorrhea of college athletes in relation to age of onset of training. *J.A.M.A.*, **246**, 1559.

FRISCH, R.E., WYSHAK, G. & VINCENT, L. (1980). Delayed menarche and amenorrhea in ballet dancers. *New Engl. J. Med.*, **303**, 17.

GAMBRELL, R. JR. (1983). Oral contraceptives and breast cancer. *Lancet*, **ii**, 1201.

GRODIN, J.M., SIITERI, P.K. & MACDONALD, P.C. (1973). Source of estrogen production in postmenopausal women. *J. Clin. Endocrinol. Metab.*, **36**, 207.

HELMRICH, S.P., SHAPIRO, S. & ROSENBERG, L. & 11 others. (1983). Risk factors for breast cancer. *Am. J. Epidemiol.*, **117**, 35.

HILL, P., GARBACZEWSKI, L., HELMAN, P., HUSKINSSON, J., SPORANGISA, E. & WYNDER, E.L. (1980). Diet, lifestyle, and menstrual activity. *Am. J. Clin. Nutr.*, **33**, 1192.

JICK, H., WATKINS, R.N. & HUNTER, J.R. & 4 others. (1979). Replacement estrogens and endometrial cancer. *New Engl. J. Med.*, **300**, 218.

MACMAHON, B., COLE, P. & BROWN, J. (1973). Etiology of human breast cancer: A review. *J. Natl Cancer Inst.*, **50**, 21.

MIETTINEN, O.S. (1976). Estimability and estimation in case referent studies. *Am. J. Epidemiol.*, **103**, 226.

MILLER, A.B. & BULBROOK, R.D. (1980). The epidemiology and etiology of breast cancer. *New Engl. J. Med.*, **303**, 1246.

MOORE, J.W., CLARK, G.M.G. & BULBROOK, R.D. *et al.* (1982). Serum concentrations of total and non-protein bound estradiol in patients with breast cancer and in normal controls. *Int. J. Cancer*, **29**, 17.

NATIONAL RESEARCH COUNCIL, (1982*a*). Diet, Nutrition, and Cancer (Washington: National Academy Press), Chap. 2, p. 7.

NATIONAL RESEARCH COUNCIL, (1982*b*). Diet, Nutrition, and Cancer, (Washington: National Academy Press), Chap. 16, p. 8.

NATIONAL RESEARCH COUNCIL. (1982*c*). Diet, Nutrition, and Cancer (Washington: National Academy Press), Chap. 5, p. 1.

PIKE, M.C., HENDERSON, B.E. & CASAGRANDE, J.T. (1981). In: *Banbury Report 8, Hormones and Breast Cancer*, Pike *et al.* (eds) p. 3. Cold Spring Harbor Laboratory.

PIKE, M.C., KRAILO, M.D., HENDERSON, B.E. & DUKE, A. (1983). Breast cancer in young women and use of oral contraceptives: Possible modifying effect of formulation and age at use. *Lancet*, **ii**, 926.

SCHNEIDER, J., KINNE, D. & FRACCHIA, A. & 4 others. (1982). Abnormal oxidative metabolism of estradiol in women with breast cancer. *Proc. Natl Acad. Sci.*, **79**, 3047.

SHERMAN, B., WALLACE, R., BEAN, J. & SCHLABAUGH, L. (1981). Relationship of body weight to menarcheal and menopausal age: Implications for breast cancer risk. *J. Clin. Endocrinol. Metab.*, **52**, 488.

SIITERI, P.K. (1981). Extraglandular estrogen formation and serum binding of estradiol: Relationship to cancer. *J. Endocrinol.*, **89**, 119P.

WARREN, M.P. (1980). The effects of exercise on pubertal progression and reproductive function in girls. *J. Clin. Endocrinol. Metab.*, **51**, 1150.

WINKELSTEIN, W. JR., SHILLITOE, E.J., BRAND, R. & JOHNSON, K.K. (1984). Further comments on cancer of the uterine cervix, smoking, and herpes virus infection. *Am. J. Epidemiol.*, **119**, 1.

WYSHAK, G., FRISCH, R.E., ALBRIGHT, N., ALBRIGHT, T. & SCHIFF, I. (1984). Lower prevalence of benign diseases of the breast and benign tumors of the reproductive system among former college athletes compared to non-athletes. 7th International Congress of Endocrinology, (Excerpta Medica, Amsterdam), p. 154.

YOUNG, J.L., JR., PERCY, C.L. & ASIRE, A.J. (1981*a*). Surveillance, epidemiology and end results, incidence and mortality data, 1973–1977 *Natl Cancer Inst. Monogr.*, **57**, 100.

YOUNG, J.L. JR., PERCY, C.L. & ASIRE, A.J. (1981*b*). Surveillance, epidemiology and end results, incidence and mortality data, 1973–1977. *Natl Cancer Inst. Monogr.*, **57**, 202.